# Developing DELPHI expert consensus rules for a digital twin model of acute stroke care in the neuro critical care unit

**DOI:** 10.1186/s12883-023-03192-9

**Published:** 2023-04-22

**Authors:** Johnny Dang, Amos Lal, Amy Montgomery, Laure Flurin, John Litell, Ognjen Gajic, Alejandro Rabinstein, on behalf of The Digital Twin Platform for education, research, and healthcare delivery investigator group, Anna Cervantes-Arslanian, Chris Marcellino, Chris Robinson, Christopher L. Kramer, David W. Freeman, David Y. Hwang, Edward Manno, Eelco Wijdicks, Jason Siegel, Jennifer Fugate, Joao A. Gomes, Joseph Burns, Kevin Gobeske, Maximiliano Hawkes, Philippe Couillard, Sara Hocker, Sudhir Datar, Tia Chakraborty

**Affiliations:** 1 Department of Neurology, Cleveland Clinic, Cleveland, USA; 2 Division of Pulmonary and Critical Care Medicine, Mayo Clinic, Rochester, USA; 3 Department of Medicine, Mayo Clinic, Rochester, USA; 4 Infectious Diseases Research Laboratory, Mayo Clinic, Rochester, USA; 5 Department of Critical Care, University Hospital of Guadeloupe, Guadeloupe, France; 6 Abbott Northwestern Emergency Critical Care, Minneapolis, USA; 7 Department of Neurology, Mayo Clinic, Rochester, USA

**Keywords:** Neuro Critical Care, AI, DELPHI, Expert Consensus, Acute Ischemic Stroke, Digital Twin

## Abstract

**Introduction:**

Digital twins, a form of artificial intelligence, are virtual representations of the physical world. In the past 20 years, digital twins have been utilized to track wind turbines' operations, monitor spacecraft's status, and even create a model of the Earth for climate research. While digital twins hold much promise for the neurocritical care unit, the question remains on how to best establish the rules that govern these models. This model will expand on our group’s existing digital twin model for the treatment of sepsis.

**Methods:**

The authors of this project collaborated to create a Direct Acyclic Graph (DAG) and an initial series of 20 DELPHI statements, each with six accompanying sub-statements that captured the pathophysiology surrounding the management of acute ischemic strokes in the practice of Neurocritical Care (NCC). Agreement from a panel of 18 experts in the field of NCC was collected through a 7-point Likert scale with consensus defined a-priori by ≥ 80% selection of a 6 (“agree”) or 7 (“strongly agree”). The endpoint of the study was defined as the completion of three separate rounds of DELPHI consensus. DELPHI statements that had met consensus would not be included in subsequent rounds of DELPHI consensus. The authors refined DELPHI statements that did not reach consensus with the guidance of de-identified expert comments for subsequent rounds of DELPHI. All DELPHI statements that reached consensus by the end of three rounds of DELPHI consensus would go on to be used to inform the construction of the digital twin model.

**Results:**

After the completion of three rounds of DELPHI, 93 (77.5%) statements reached consensus, 11 (9.2%) statements were excluded, and 16 (13.3%) statements did not reach a consensus of the original 120 DELPHI statements.

**Conclusion:**

This descriptive study demonstrates the use of the DELPHI process to generate consensus among experts and establish a set of rules for the development of a digital twin model for use in the neurologic ICU. Compared to associative models of AI, which develop rules based on finding associations in datasets, digital twin AI created by the DELPHI process are easily interpretable models based on a current understanding of underlying physiology.

## Background

Artificial intelligence is a broad term that encompasses any computational system that can perform the functions that make people seem intelligent [1]. Artificial intelligence has become omnipresent in our daily lives through personal assistants, facial recognition, automated cars, and more [2]. This technology has also started to find its place in healthcare. In the field of cardiology, FDA-cleared and clinically applied artificial intelligence models already exist to predict fractional flow reserve and map out electrical heart activity from body surface potentials [3]. For diseases such as multiple sclerosis, which often has a heterogeneous course and where evaluation requires integration of multidimensional data including clinical assessment, imaging, electrophysiology, and biomarkers, preliminary models are being developed to aid diagnosis and rehabilitation of these patients, leading to a new era of individualized healthcare [4].

Digital twins, a form of artificial intelligence, are virtual representations of the physical world [5]. In the past 20 years, the digital twin concept has been utilized in other fields to track wind turbines' operations, monitor spacecraft's status, and even create a model of the Earth for climate research [6, 7]. The Archimedes model paved the way for digital twins in health care by predicting individual diabetes risk, creating a representation of the physiology of diabetes, and modeling the effects of treatments and complications [8, 9]. During the height of the COVID-19 pandemic, digital twins allowed healthcare providers to model the effects of various drugs on the individual level and model the spread of the disease on an organizational and population level [10]. Digital twins are also starting to play a role in educating the next generation of physicians through interactive simulation platforms such as JustPhysiology and HumMod [11, 12].

In the neurocritical care unit, artificial intelligence has been used to help interpret continuous EEGs, monitor ICP waveforms, triage CT scans, identify extracellular proteins of cerebral ischemia, predict the risk of hemorrhagic transformation, and prognosticate recovery [13–16]. With the rise of multimodal monitoring in the Neuro ICU, there is an increased demand for interpreting and making sense of the influx of multidimensional data [17]. Despite the advances made in the field of NCC so far, little research has been done regarding the applications of digital twins to the neurocritical care unit. As we develop new ways to monitor patient physiology, healthcare is entering an era of "big data," and artificial intelligence, particularly digital twins, is an emerging technology that physicians are looking to make sense of this vast amount of data [18, 19].

While artificial intelligence holds much promise for clinicians working in Neurocritical Care (NCC), the question remains on how to build these models best. Associative AI models rely on drawing associations and identifying patterns from large data sets to make recommendations. As evidenced by the failure of IBM’s Watson, these models are limited by the data sets they are trained on, and how these models reach their conclusions often needs to be more apparent to clinicians [20].

Compared to associative AI models, causal AI models are based on understanding underlying physiological variables and causal pathways [21]. Creating a causal AI requires a foundation of expert rules that define the interaction between variables, connect concepts through Bayesian networks, and model how different interventions and interactions affect changes in various organ systems as reflected by clinical markers such as vitals, physiological signs, and biomarkers [22]. This foundational model is subsequently trained and refined on prospective clinical data.

DELPHI is a method used particularly in healthcare to systematically bring together knowledge, creating consensus amongst experts within a field, and is one way to establish these expert rules [23]. During the COVID-19 pandemic, a DELPHI process was used to gain expert consensus for the best management practices of patients with COVID-related acute respiratory failure [24]. Key points to consider when developing DELPHI consensus include defining a-priori, the threshold for consensus, how consensus will be defined, and the criteria for concluding the DELPHI process, such as after a certain number of rounds [25–27].

Our group has previously created a digital twin model to predict acute response to the treatment of sepsis. It has identified the potential for applying such models to augment clinical education and potentially clinical decision-making in the field of NCC [28, 29]. This project expands on our previous work by demonstrating the methodical use of DELPHI consensus to establish a foundational set of expert rules for use in developing a similar causal digital twin model of acute ischemic stroke in the Neuro Critical Care unit that will be based on a transparent mechanistic understanding of underlying pathophysiology. Similar work has been done for other organ systems [30, 31].

## Methods

### Model conception

**Fig. 1:**
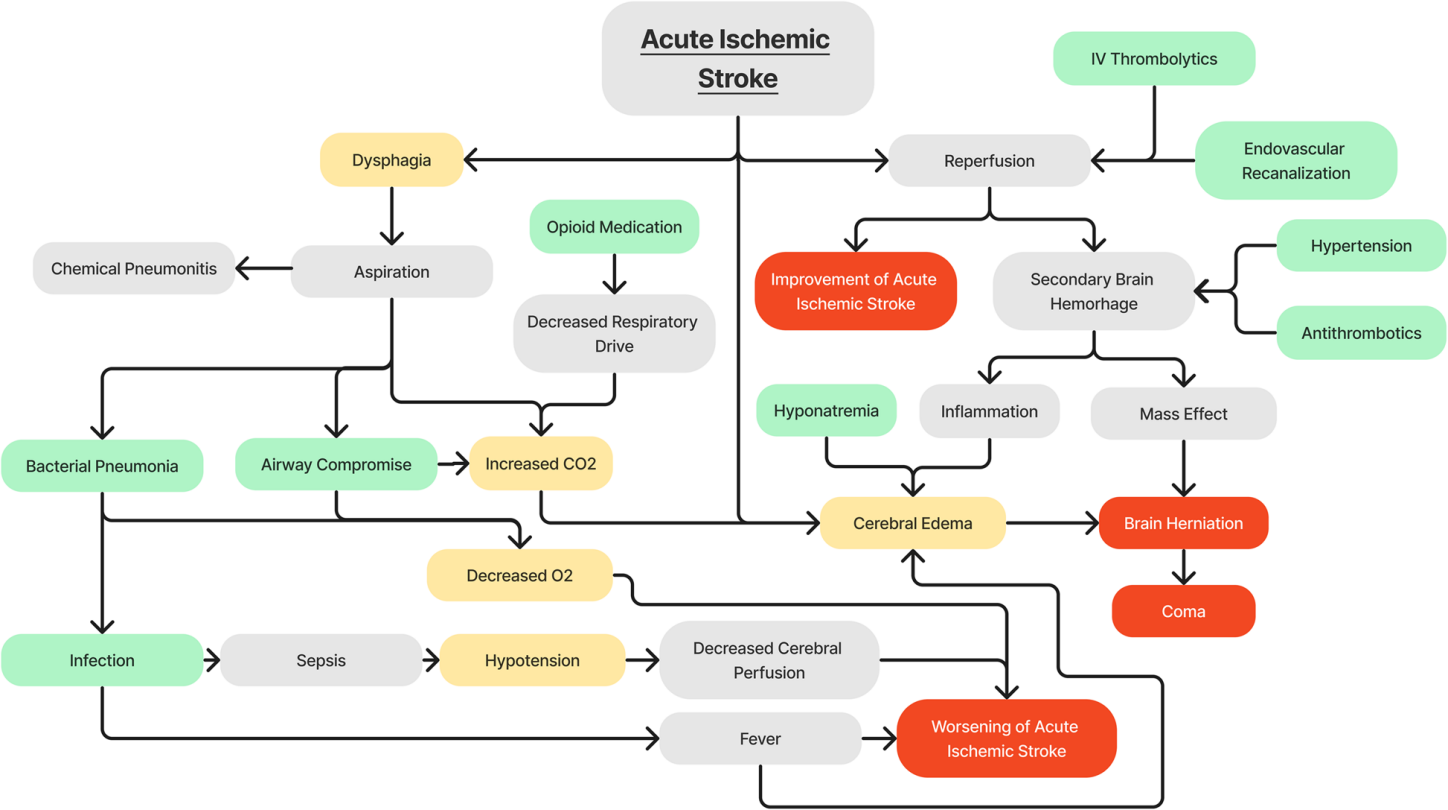
Directed Acyclic Graph (DAG) providing a visual representation of connections between different concepts and variables. Modifiable variables are represented in green, semi-modifiable variables in yellow, intermediary states in gray, and end states in red. These nodes are connected by unidirectional black arrows depicting the flow of processes from one condition to the subsequent state

**Fig. 2:**
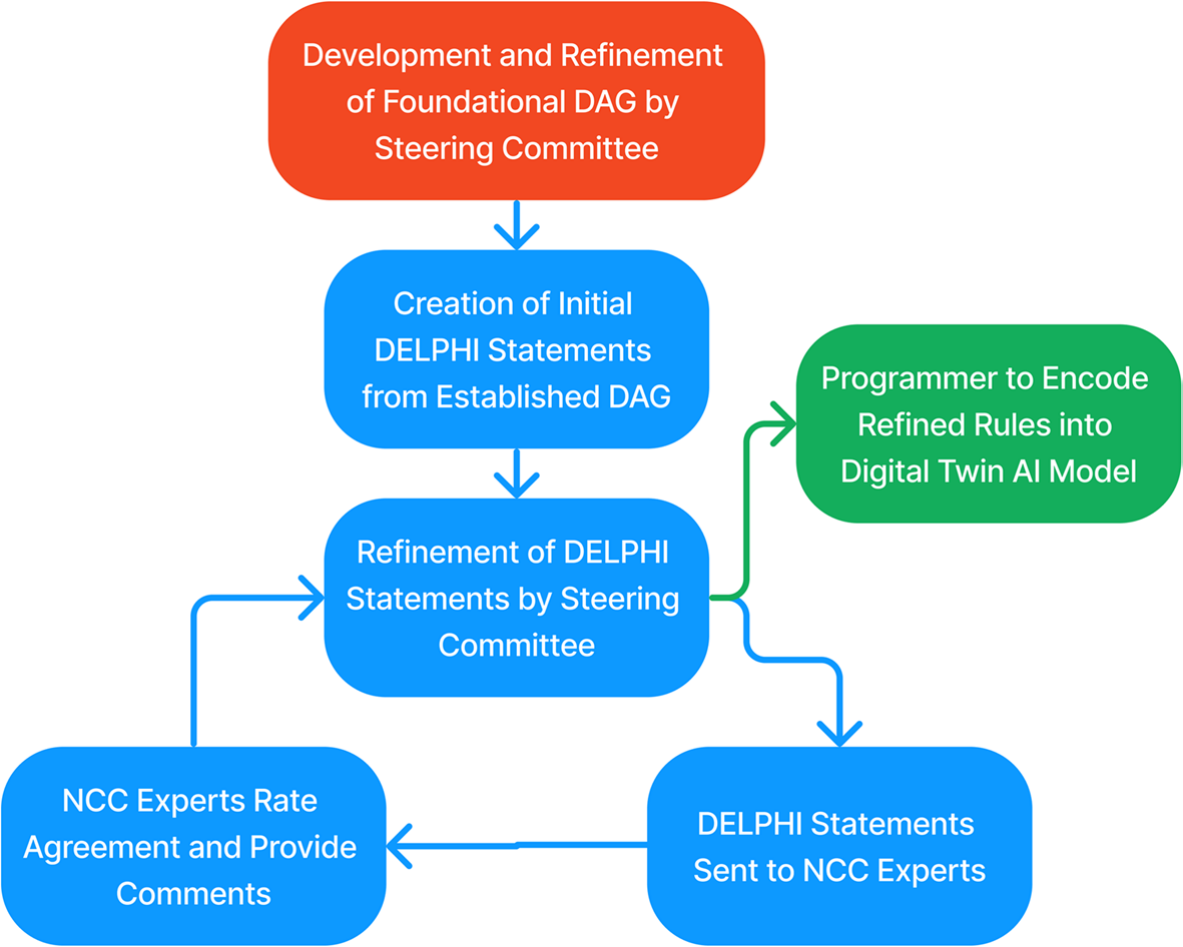
Flowchart providing an overview of the DELPHI consensus process. A foundational Directed Acyclic Graph (DAG) model is first constructed and refined. From this model, DELPHI statements are established, sent to Neurocritical Care (NCC) experts, and further refined before being deemed valid and sent to a programmers to incorporate into the Digital Twin AI Model

An initial steering committee of clinicians from the fields of neurology, neurocritical care (NCC), emergency medicine, and pulmonary critical care medicine drafted an initial model of the pathophysiology and management of acute ischemic stroke through a Directed Acyclic Graph (DAG) with concepts being connected by Bayesian networks (Fig. 1). This conceptual model was iteratively revised and refined by the steering committee until deemed ready to be translated into DELPHI consensus statements. From the DAG, 20 main DELPHI consensus statements, each with six sub-statements, were created and further refined for use in the first round of DELPHI consensus (Fig. 2).

### Surveys

This is a descriptive study where experts were invited to participate in this DELPHI process keeping in mind the requisites of diversity in sex (males and females), years of experience (ranging from 1 to 30 years of experience), varied topics of interest within the subspecialty, and geographical area of clinical practice. Upon acceptance, a modified Delphi panel of 23 NCC experts was established. These NCC experts were invited by email to participate in three rounds of DELPHI consensus statements. Three experts did not respond to the initial call to participate in the DELPHI process, and two provided incomplete responses to the initial survey round (and therefore excluded). In total, 18 Neuro Critical Care experts participated and completed all three rounds of DELPHI consensus. The steering committee did not participate in the surveys but would meet between each round to revise DELPHI statements. Survey responses were collected through a secure REDCap form through the Mayo Clinic platform, and surveys were sent out through a secure email link. REDCap data were de-identified and then analyzed in third-party spreadsheet software.

### DELPHI statements

The initial DELPHI survey consisted of 20 main statements, each with six sub-statements making for 120 statements total. A 7-point balanced Likert scale measured agreement with each sub-statement. Sub-statements included direction statements, generally defining how variables interacted; probability, timing, and intensity statements, defining how likely, when, and how much variables interacted; and therapeutic impact and contingency statements, clarifying the effects of the intervention on the interactions and contingent situations where the interaction would occur differently if at all. Additionally, each main statement had an optional free text area where experts could clarify their thought process and provide recommendations for further refining the DELPHI statements in ways that a balanced Likert Scale could not capture.

### Consensus

Consensus was defined as a-priori as greater than or equal to 80% of participants responding with a 6 (“Agree”) or 7 (“Strongly Agree”) in a 7-point Likert scale for any given statement [25]. In between each round of DELPHI consensus, statements were edited by the steering committee. Statements that had reached consensus on previous rounds of DELPHI were not included in later DELPHI rounds. Statements that did not reach consensus (< 80% agreement) were reviewed and revised by the steering committee, and expert comments were incorporated for the next round of DELPHI. The endpoint of the study was defined as the completion of three separate rounds of DELPHI consensus. All DELPHI statements that reached consensus by the end of three rounds of DELPHI consensus would go on to be used to inform the construction of the digital twin model.

### From DELPHI statement to digital twin

DELPHI statements provide the core foundation of knowledge upon which the Digital Twin model will be constructed. This Digital Twin model, created with the assistance of a programmer, will consist of Bayesian networks, where nodes, representing quantities, variables, or states, are interconnected with other nodes [11, 32]. The probabilistic interactions between these nodes, based on causal effect and prior knowledge, can be visualized in a Directed Acyclic Graph (Fig. 1). These digital twin models are then refined through prospective observation in an actual critical care setting, where the predictions of the model are measured against patient outcomes to assess agreement between the model and what is seen in clinical practice [28].

## Results

**Fig. 3:**
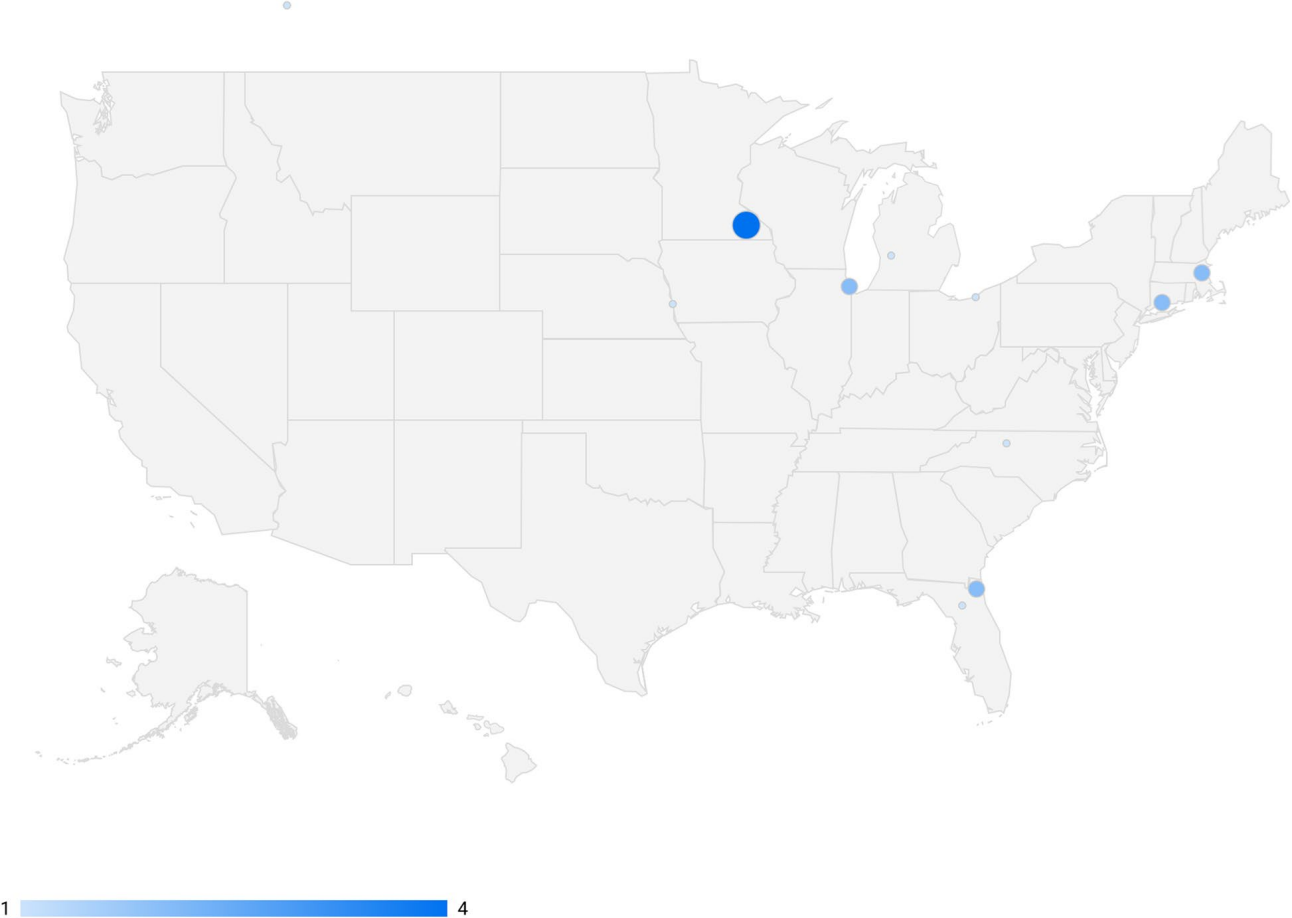
Map of the geographic distribution of Neurocritical Care Experts

Three rounds of DELPHI consensus were completed from February 2022 to July 2022. Of the 18 experts participating in the DELPHI consensus process, 14 (77.8%) were male and 4 (23.2%) were female. 17 (94.7%) were from the United States across nine different states, and 1 (5.3%) was from Canada (Fig. 3). Experts ranged from 1 to 30 years in practice with an average of 9.78 years and a standard deviation of 7.58 years. NCC experts identified additional interests in vascular neurology, traumatic brain injury, intracranial hemorrhage, and seizure, among others.

**Fig. 4:**
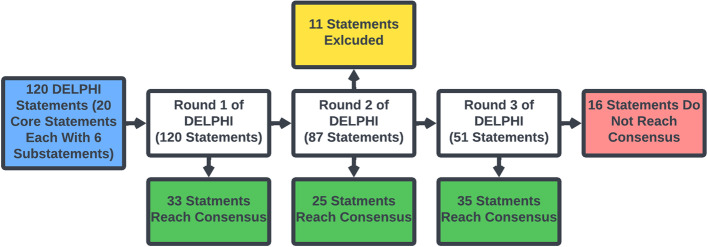
Flow chart of the DELPHI consensus process. After three rounds of DELPHI consensus, 93 statements reached consensus (green), 11 statements were excluded (yellow), and 16 statements did not reach consensus (red)

**Fig. 5:**
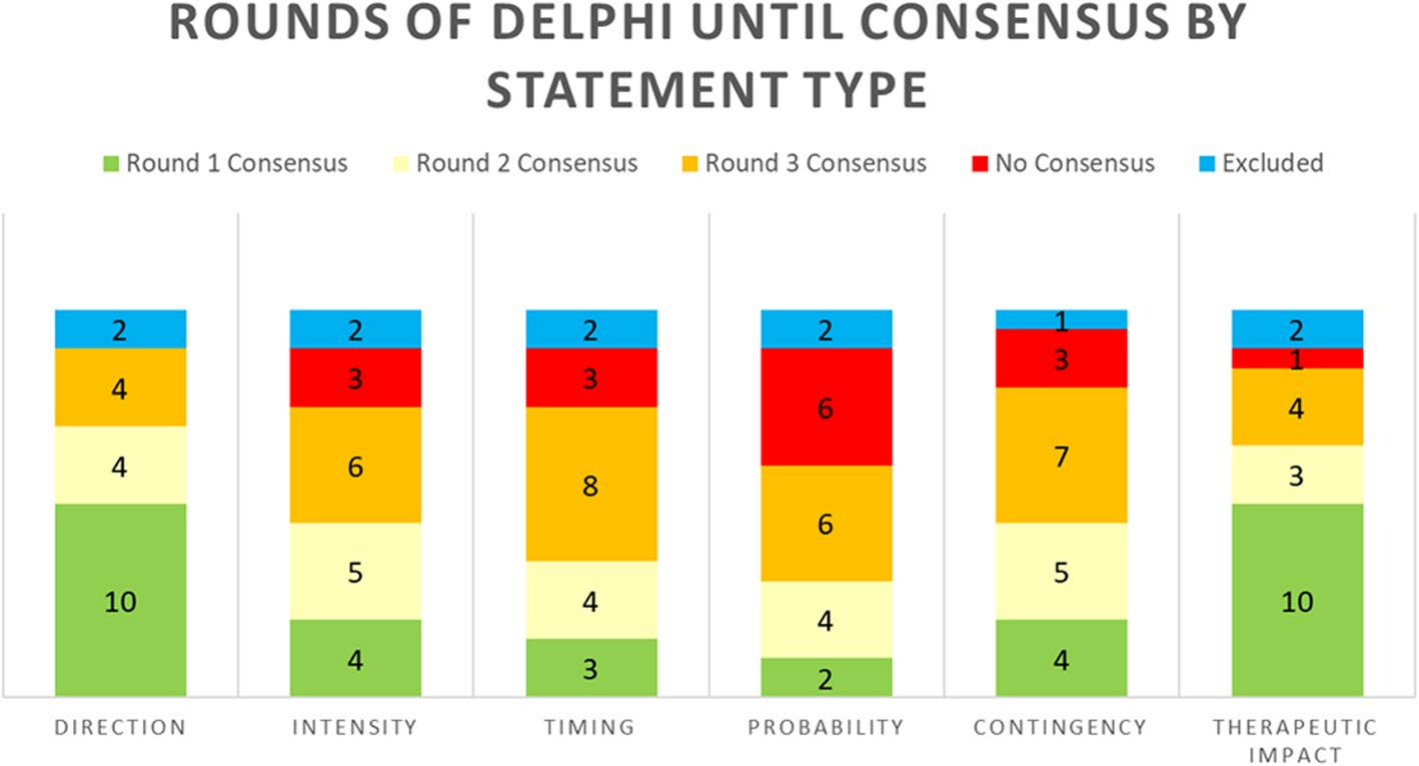
Stacked bar graph demonstrating how many rounds of DELPHI were needed to reach consensus by sub-statement type

Of the original 120 DELPHI statements, 93 (77.5%) statements reached consensus, 11 (9.2%) statements were excluded, and 16 (13.3%) statements did not reach consensus after three rounds of DELPHI (Fig. 4). 33 (27.5%) statements reached consensus after the first round of DELPHI, 25 (20.8%) statements reached consensus after the second round of DELPHI, and 35 (29.2%) statements reached consensus after the third round of DELPHI (Table 1). Of the 33 statements that reached consensus in the first round, 10 (30.3%) statements were direction or therapeutic impact statements each, 4 (12.1%) statements were intensity or contingency statements each, 3 (9.0%) statements were timing statements, and 2 (6.1%) statements were probability statements. Of the 16 statements that did not reach consensus, 6 (37.5%) statements were probability statements, 3 (18.8%) statements were intensity, timing, or contingency statements each, 1 (6.3%) statement was a therapeutic impact statement, and no statements were direction statements (Fig. 5).

**Table 1:** Final table of DELPHI statements accompanied by which round of DELPHI reached consensus

Statement	Rounds Until Consensus
Direction: Hypotension worsens acute ischemic stroke	1
Intensity: High (Increased effect of hypotension on worsening of acute ischemic stroke with large vessel occlusion and increased penumbral size)	2
Timing: Immediate (1–4 h)	2
Probability: High	2
Contingency: Hypotension will have a larger impact on worsening ischemic stroke size in large vessel occlusion than in small vessel/lacunar infarcts. Effect of hypotension is pronounced with increased stroke penumbra	2
Therapeutic Impact: Providers should avoid iatrogenic hypotension in patients with acute ischemic stroke	2
Direction: Aspiration leads to chemical pneumonitis or bacterial pneumonia	1
Intensity: Moderate (Modulated by severity of resulting pneumonia)	3
Timing: Acute-Subacute (Hours to days. Clinical and radiographic evidence of pneumonia may be delayed.)	3
Probability: Moderate (Probability modulated by volume and frequency of aspiration events as well as stroke type. Patients may still have micro-aspiration in the absence of macro-aspiration	3
Contingencies: Increased risk of aspiration with impaired consciousness, speech or swallow	1
Therapeutic Impact: Preventing large aspiration events can prevent chemical pneumonitis or pneumonia	2
Direction: Acute ischemic stroke impairs swallowing and compromises airways	1
Intensity: High (Aspiration events and compromised airways can be life-threatening)	3
Timing: Immediate-Subacute (Hours to Days)	3
Probability: High (Depending on location of infarct and presence of oral-pharyngeal-laryngeal dysfunction)	3
Contingencies: Stroke leading to impaired swallowing depending on which area of the CNS has been affected by the stroke	1
Therapeutic Impact: Swallow studies before reinitiating a diet can prevent aspiration	1
Direction: Decreased GCS leads to impairment of airway patency, ventilatory impairment, and respiratory failure	Excluded
Intensity: High	Excluded
Timing: Subacute (12–24 h)	Excluded
Probability: High	Excluded
Contingencies: Increased risk of aspiration with neurologic diseases or impaired consciousness	1
Therapeutic Impact: Preventing aspiration (oral care, feeding in semi recumbent position, swallow evaluations) reduces risk of compromised airway	Excluded
Direction: Reperfusion of ischemic stroke can lead to improvement of stroke	1
Intensity: High	1
Timing: Acute (4–12 h)	2
Probability: High	2
Contingencies: Patients should not receive thrombolytics or endovascular recanalization if contraindicated. Patients may still receive thrombolytics past the indicated time frame (3–4.5 h for thrombolysis and 24 h for endovascular recanalization since symptom onset) if benefits (based on perfusion studies and location/severity of stroke) outweigh the risks	3
Therapeutic Impact: Timely administration of thrombolytics can lead to improvement of outcomes in ischemic stroke	1
Direction: Infection can lead to low blood pressures	3
Intensity: Variable (Depends on severity of infection)	3
Timing: Bimodal with Acute and Subacute Groups	2
Probability: Moderate (Not every infection will lead to a systemic response)	3
Contingencies: Higher incidence in infants and elderly as well as those predisposed to infections (i.e., immunocompromised)	1
Therapeutic Impact: Antibiotics and source control procedures can be used to treat infection and vasopressors can be used to maintain blood pressures	1
Direction: Opioid medications can decrease minute ventilation	1
Intensity: High. Dose dependent. Increased effect with increasing dose	1
Timing: Immediate-Acute (1–24 Hours to onset)	3
Probability: Medium (Dose dependent)	2
Contingencies: Opioids should be used with caution in patients with renal dysfunction, chronic hypercapnic respiratory failure, who are opioid naïve, and who are elderly	2
Therapeutic Impact: Careful administration of opioids can prevent respiratory depression. Naloxone can reverse the effects of opioid medications. Intubation and mechanical ventilation can maintain adequate minute ventilation	1
Direction: Hypertension can increase risk of secondary (post-stroke) brain hemorrhage in patients who have received tPA or thrombectomy or who have a coagulopathy	Excluded
Intensity: High	Excluded
Timing: Subacute (12–24 h)	Excluded
Probability: High. (For patients who have received tPA, thrombectomy, or are coagulopathic)	Excluded
Contingencies: More common in patients with age-related vasculopathy or CAA and patients undergoing reperfusion	Excluded
Therapeutic Impact: Control of high blood pressure with medications can reduce risk of secondary brain hemorrhage	Excluded
Direction: Administration of thrombolytics can lead to brain hemorrhage	2
Intensity: High	2
Timing: Immediate-Subacute (1–24 Hours. Highest risk in the acute period, but still possible in the subacute period.)	3
Probability: Low (Increased with later time of onset and increased NIHSS severity)	3
Contingencies: Use of thrombolytics even when indicated increases risk for brain hemorrhage	2
Therapeutic Impact: While antithrombotics have a risk of brain hemorrhage, the benefits in treatment of acute ischemic stroke must be weighed against the risks of hemorrhage	1
Direction: Reperfusion can lead to potential reperfusion injury with secondary brain hemorrhage	3
Intensity: High (Intensity depends on degree of brain hemorrhage and subsequent edema.)	No Consensus
Timing: Immediate-Acute (Onset within 24 Hours)	3
Probability: Low (Depends on presence of contingency such as duration of vascular compromise, time to reperfusion, coagulopathy, degree of hypertension, and quality of collaterals)	No Consensus
Contingencies: Degree of reperfusion injury depends on duration of vascular compromise, time to reperfusion, degree of hypertension, quality of collaterals	2
Therapeutic Impact: Brain edema and secondary hemorrhage are complications after reperfusion of acute ischemic stroke and patients should be monitored for these complications	1
Direction: Secondary brain hemorrhage causes brain edema	1
Intensity: High (Brain edema, when it occurs, can lead to subsequent brain herniation)	3
Timing: Subacute	1
Probability: High (Dependent on size of initial stroke and size of subsequent hemorrhage)	No Consensus
Contingencies: Level of inflammation and edema is dependent on size of initial stroke and size of subsequent hemorrhage. Other contingencies are being researched	3
Therapeutic Impact: Brain edema must be properly managed with osmotherapy or decompressive surgery to prevent subsequent brain herniation	3
Direction: Secondary brain hemorrhage causes mass effect	1
Intensity: High (Mass effect, when it occurs, can lead to subsequent brain herniation)	3
Timing: Immediate—Subacute (Bimodal distribution with initial mass effect occurring immediately as well as subacutely from resulting edema after a few days.)	No Consensus
Probability: Moderate (Dependent on hemorrhage size, location, and subsequent edema)	3
Contingencies: Degree of mass effect depends on ICH volume and location as well as degree of subsequent edema. Degree of mass effect is decreased in patients with brain atrophy	3
Therapeutic Impact: Mass effect must be properly managed with osmotherapy or decompressive surgery to prevent subsequent brain herniation	3
Direction: Brain edema and mass effect lead to brain herniation and coma	1
Intensity: High	2
Timing: Acute—Subacute (Hours to days for peak swelling)	3
Probability: Moderate	2
Contingencies: Brain edema and mass effect over a certain level lead to increased risk of brain herniation. Brain herniation may not necessarily be correlated with increased ICP. Degree of brain edema and mass effect is increased by increased mass lesion size, increased pressure gradient, and temporal location	2
Therapeutic Impact: Brain edema and mass effect can cause subsequent brain herniation. Serial neurologic exams and neuroimaging may provide an early warning to clinicians. ICP and/or brain edema can be reduced by sedation (decreasing brain metabolism), analgesia, elevating the head of the bed, hyperventilation, hypertonic saline, CSF drainage, and decompressive hemicraniectomy	3
Direction: Fever can lead to worsening of acute ischemic stroke	1
Intensity: Moderate	1
Timing: Acute-Subacute (Hours to Days from onset)	3
Probability: Moderate	No Consensus
Contingencies: Clinical worsening of acute ischemic stroke and level of neurologic deficit correlate with degree of fever	No Consensus
Therapeutic Impact: Preventing infection can curb the development of fever in patients with acute ischemic stroke. Fever is also a known phenomenon resulting from ischemic stroke and is thought to be a negative sign for prognosis. Hyperthermia should be identified and treated	1
Direction: Acute hyponatremia worsens brain edema	2
Intensity: High (Degree of edema depends on magnitude and rate of change of serum sodium concentration.)	3
Timing: Subacute (May be hours to days until edema is clinically significant)	No Consensus
Probability: High	No Consensus
Contingencies: Significance of hyponatremia on brain edema is mostly determined by severity/magnitude and rate of change of serum sodium concentration. Providers should identify the primary cause of hyponatremia	No Consensus
Therapeutic Impact: Brain edema due to acute hyponatremia can be reduced with hypertonic saline. Relative reduction is serum sodium should be prevented in patients with chronic hyponatremia	2
Direction: Hypercapnia increases ICP	2
Intensity: High	2
Timing: Immediate	1
Probability: High	3
Contingencies: Degree of increased ICP usually correlates with increased with rate of and magnitude of CO2 increase. ICP increase may not be sustained. Effect of hypercapnia is modulated by baseline CO2 level of patient and may be modulated by presence of COPD, obesity, or hypoventilation syndrome	3
Therapeutic Impact: Patients can be transiently hyperventilated to reduce elevatedCO2 and reduce brain edema. This is only a bridge to definitive treatment. Ventilation should be monitored by occasionally checking PCO2 in mechanically ventilated patients (especially with changes in sedation or with fever)	1
Direction: Ischemic stroke leads to cerebral edema	1
Intensity: Moderate-High (Depends on severity of stroke)	1
Timing: Subacute	1
Probability: Moderate-High (Depends on severity of stroke)	1
Contingencies: Degree of cerebral edema is dependent on severity of stroke. Subsequent mass effect and clinical effects are modulated by degree of cerebral atrophy	3
Therapeutic Impact: Cerebral edema should be managed with osmotic therapy ± a brief period of hyperventilation to prevent devastating brain herniation. Decompressive surgery helps to relieve cerebral edema	No Consensus
Direction: Acute ischemic stroke can lead to secondary brain hemorrhage	2
Intensity: Variable	2
Timing: Variable	2
Probability: Low	1
Contingencies: Degree of secondary brain hemorrhage increases with increased size and severity of stroke, increased blood pressure, cardioembolic stroke, intrinsic coagulopathy, and early use of antithrombotics	No Consensus
Therapeutic Impact: Acute ischemic stroke may undergo hemorrhagic transformation leading to secondary brain hemorrhage	1
Direction: Sepsis (Septic Shock) causes supply–demand mismatch in oxygenation	3
Intensity: Moderate (Intensity of supply–demand mismatch depends on severity of sepsis.)	No Consensus
Timing: Subacute (Hours to days to onset)	No Consensus
Probability: Moderate (Probability of supply–demand mismatch depends on severity of sepsis.)	No Consensus
Contingencies: Degree of resulting supply–demand mismatch is modified by source and severity of infection as well as premorbid conditions of patients	3
Therapeutic Impact: Treatment of infection with antibiotics can help manage resulting shock state and reduce supply–demand mismatch. Supplemental oxygenation can help restore oxygenation	3
Direction: Decreased oxygenation levels can cause tissue hypoxia and lead to worsening of acute ischemic stroke	3
Intensity: Moderate (Intensity of supply–demand mismatch depends on severity of sepsis.)	No Consensus
Timing: Acute-Subacute (Hours to Days to onset)	3
Probability: Moderate (Probability of supply–demand mismatch depends on severity of sepsis.)	No Consensus
Contingencies: Worsening of acute ischemic stroke and neurologic deficits is modulated by the degree and duration of hypoxemia	3
Therapeutic Impact: Supplemental oxygenation can help restore oxygenation. Intubation and mechanical ventilation may be necessary if respiratory drive or airways are compromised	1

Two sets of statements were excluded after the second round of DELPHI. One set of statements, “Decreased GCS leads to impairment of airway patency, ventilatory impairment, and respiratory failure.” was excluded due to redundancy to the statements preceding it, “Acute ischemic stroke impairs swallowing and compromises airways.”. The second set of statements, “Hypertension can increase risk of secondary (post-stroke) brain hemorrhage in patients who have received tPA or thrombectomy or who have a coagulopathy.” was excluded due to contention between experts regarding the mixed evidence on the topic.

## Discussion

We report the application of a structured DELPHI process with multiple iterative rounds to generate consensus among experts in the field of NCC on a series of expert rules that will act as a foundation for the creation of a digital twin artificial intelligence model specifically designed to simulate the acute clinical course of ischemic stroke in the critical care setting. Incorporating NCC experts' knowledge and real-world clinical experience, this digital twin model will further be refined prospectively with real-time patient data [28]. This model will also be incorporated into an existing digital twin model of sepsis and future models of other organ systems under development by our research group.

In the first round of DELPHI, statements that garnered the highest amount of agreement included “Stroke leads to impaired swallowing depending on which area of the CNS has been affected by the stroke.”, “Reperfusion of ischemic stroke can lead to improvement of stroke.”, and “Antibiotics and source control procedures can be used to treat infection and vasopressors can be used to maintain blood pressures.”, each with 100% consensus. Although these statements appear relatively obvious, it was still essential to include these statements.

While NCC experts generally agreed on the overarching direction statements, there was significant disagreement regarding the nuances of these interactions' intensity, timing, probability, and contingencies. For example, when the factors of large vessel occlusion and penumbral size were introduced to the intensity statement of "Hypotension worsens acute ischemic stroke." consensus increased from 78 to 100%. Similarly, when the severity of infection was incorporated into the intensity statement of "Infection can lead to low blood pressure." consensus increased from 56 to 100%. While experts agreed with all main direction statements by the end of three rounds of DELPHI, areas of disagreement persisted relating to the details of when these interactions occurred, how intense those interactions are, how likely those interactions are to happen, and the contingent situations where these interactions may not always be accurate.

Expert comments identified interactions that should have been considered during the initial creation of the DELPHI statements and highlighted the importance of clinical experience in developing these models. For example, when looking at the contingencies related to the effect of hypercapnia on ICP, the statement evolved from “None.” (56% Consensus) to “Degree of increased ICP is increased with a rate of and magnitude of CO2 increase.” (78% Consensus) to “Degree of increased ICP usually correlates with increased with a rate of and magnitude of CO2 increase. ICP increase may not be sustained. The effect of hypercapnia is modulated by the baseline CO2 level of the patient and may be modulated by the presence of COPD, obesity, or hypoventilation syndrome.” (94% Consensus).

Some reasons for statements not reaching consensus included disagreement on particular words or phrasing, inability to capture the full nuance of clinical scenarios (particularly with intensity, timing, and probability statements), and uncertainty in the literature. For example, when looking at the therapeutic impacts of cerebral edema, the statement evolved from “Elevated intracranial pressure secondary to edema should be managed with osmotic therapy ± a brief period of hyperventilation to prevent devastating brain herniation.” (50% consensus) to “Cerebral edema should be managed with osmotic therapy ± a brief period of hyperventilation to prevent devastating brain herniation. Hemicraniectomy acts as a definitive treatment to relieve cerebral edema.” (61% consensus) to “Cerebral edema should be managed with osmotic therapy ± a brief period of hyperventilation to prevent devastating brain herniation. Decompressive surgery helps to relieve cerebral edema.” (78% consensus). Some expert comments that guided the refinement of this statement included “[Patients] may herniate despite normal ICPs.” and “Mass effect, not necessarily elevated ICP, is what is typically being managed. The most effective treatment is hemicraniectomy.” Comments on the statement in the final DELPHI round included, "Decompressive surgery helps to prevent secondary injury caused by cerebral edema to the non-infarcted brain.” And “Edema may not always need to be treated medically.”

The results of this study demonstrated the application of a DELPHI process to establish expert consensus on foundational rules to be used in developing a digital twin model of acute ischemic stroke. While models such as Archimedes have been developed for prognostication in the chronic disease and outpatient setting, no good model exists for use in the critical care unit, particularly the neurocritical care unit [8, 9]. While emerging artificial intelligence models are currently under development, these models can be limited by a lack of transparency and reliance on artificial intelligence drawing vague associations among large data sets rather than casual relationships based on an understanding of underlying patient physiology [13].

Causal AI models, such as the one we propose, will leverage the knowledge and experiences of leading neuro intensivists cultivated over years of studying disease pathophysiology and treating real-life patients. These models, with their interactions depicted through DAGs, allow for a higher level of transparency than existing associative AI models [32]. The subsequent aim and intention are to provide the end-users (learners, bedside clinicians, educators) with a model that can clearly demonstrate the interplay between various physiologic models while clearly displaying the expert consensus statements underlying the code. Such a model has never been established for use in the neurocritical care unit. Additionally, the expert rules created from this DELPHI process will contribute to a larger project integrating knowledge from various specialties within critical care, allowing us to integrate further physiologic variables not directly addressed in this DELPHI process.

Limitations of the study include the subjective nature of survey data, limited sample size, and language restrictions. The extensive nature of the initial round of 120 DELPHI statements limited the participation of some experts and establishing consensus through DELPHI can be a time-intensive process. While a panel of 18 Neuro Critical experts is a sizable group, the study would benefit from the input of more participants, particularly from outside of the US and Canada.

Artificial intelligence is not without its limitations [13]. IBM’s Watson had big visions of integrating artificial intelligence into the healthcare industry, but promises of new insights from large data sets soon turned into frustrations with the complexity and inflexibility of the system, struggling to decipher electronic medical record data, and wasted time wrestling with the new technology rather than taking care of patients [20]. While Watson performed superbly in the testing phase, the real-world experience was largely underwhelming.

The increasing integration of artificial intelligence and healthcare will also lead to more questions of government regulation, privacy, bias, and ethics. The government will need new regulatory frameworks to monitor these novel artificial intelligence models integrated into patient care as "Software as Medical Devices" to ensure these software are safe, valid, and efficacious and respect patient privacy [33–36]. Questions also remain about the liability and biases that could come with using this new technology.[13] In a world of increasing data, artificial intelligence and digital twins, in particular, hold the promise of integrating multidimensional clinical, laboratory, genomic, biochemical, protein, and metabolic data in the healthcare field, allowing for more efficient and personalized treatment of disease. However, we must remain cognizant of the technical limitations and ethical quandaries that come with this new technology [37, 38].

Future directions include creating a proof of concept that applies these expert rules to expand on our existing digital twin system for sepsis, developing similar systems of expert rules in other organ systems (oxygenation and ventilation, inflammation, acute kidney injury, etc.), verifying the model with prospective patient data, application of the digital twin model for use in graduate medical education, and eventually integrating the technology into clinical practice. After the expert rules are incorporated into the current digital twin model, it will be essential to validate and adjust the model to real-time future iterations using EHR data from real patients to ensure the model's reliability. This digital twin model, once validated, will allow trainees to practice making decisions on an accurate and realistic model of patient physiology without putting a real patient at risk. With further development, this artificial intelligence model has the potential to be integrated with similar models of different organ systems to create a more realistic replica of a patient’s physiology and eventually develop into a clinical decision-making tool that changes how medicine will be practiced in the future.

## Conclusion

This descriptive study demonstrates the application of the DELPHI process to generate consensus among experts for the development of a “digital twin” artificial intelligence model for use in NCC. After three rounds of DELPHI, we gained consensus on 93 (77.5%) of 120 initial DELPHI statements, with 100% consensus on all main direction statements. Compared to other models that rely on “black-box” associative artificial intelligence, this proposed digital twin model exploits the *causal* AI model based on a solid foundation of expert rules and causal mechanisms. This study demonstrates one method, the DELPHI consensus method, by which a foundation of expert rules can be established. In the future, this type of model can be used as a simulation tool in graduate medical education, and after extensive validation, it could also serve as a clinical decision aid, changing how medicine will be practiced in the future.

## Data Availability

The datasets generated during and/or analyzed during the current study are available from the corresponding author on reasonable request.

## Abbreviations

NCC: Neurocritical care
AI: Artificial intelligence
DAG: Directed acyclic graph
